# Modeling the Mechanisms by Which HIV-Associated Immunosuppression Influences HPV Persistence at the Oral Mucosa

**DOI:** 10.1371/journal.pone.0168133

**Published:** 2017-01-06

**Authors:** Meghna Verma, Samantha Erwin, Vida Abedi, Raquel Hontecillas, Stefan Hoops, Andrew Leber, Josep Bassaganya-Riera, Stanca M. Ciupe

**Affiliations:** 1 Nutritional Immunology and Molecular Medicine Laboratory, Biocomplexity Institute of Virginia Tech, Blacksburg, VA, United States of America; 2 Department of Mathematics, Virginia Tech, Blacksburg, VA, United States of America; Universita Vita Salute San Raffaele, ITALY

## Abstract

Human immunodeficiency virus (HIV)-infected patients are at an increased risk of co-infection with human papilloma virus (HPV), and subsequent malignancies such as oral cancer. To determine the role of HIV-associated immune suppression on HPV persistence and pathogenesis, and to investigate the mechanisms underlying the modulation of HPV infection and oral cancer by HIV, we developed a mathematical model of HIV/HPV co-infection. Our model captures known immunological and molecular features such as impaired HPV-specific effector T helper 1 (Th1) cell responses, and enhanced HPV infection due to HIV. We used the model to determine HPV prognosis in the presence of HIV infection, and identified conditions under which HIV infection alters HPV persistence in the oral mucosa system. The model predicts that conditions leading to HPV persistence during HIV/HPV co-infection are the permissive immune environment created by HIV and molecular interactions between the two viruses. The model also determines when HPV infection continues to persist in the short run in a co-infected patient undergoing antiretroviral therapy. Lastly, the model predicts that, under efficacious antiretroviral treatment, HPV infections will decrease in the long run due to the restoration of CD4+ T cell numbers and protective immune responses.

## Introduction

Infection with the human immunodeficiency virus (HIV) afflicts over 35 million people worldwide and results in impaired immune responses which may affect defenses against other pathogens. In the absence of protective vaccines, current management of HIV consists of administration of combination antiretroviral therapy (cART)—which suppresses viral replication and, consequently, drastically reduces morbidity and mortality [1, 2]. cARTs are 99% effective; however, antiviral drug resistance (mainly caused by non-compliance) against some of the anti-HIV drugs has been reported in up to 60% of the patients [3]. Moreover, a cure for HIV is challenging due to the strict need for lifelong treatment to avoid virus rebound from activation of latent reservoirs.

Apart from the acquired immunodeficiency syndrome (AIDS), HIV increases the risk of developing opportunistic infections by other infectious agents, including viruses: papillomavirus, herpesviruses, flaviviruses; and bacteria: *Helicobacter pylori*, *Salmonella typhimurium*, *Chlamydophila pneumonia* [4]. Epidemiological data suggests that HIV patients have an increased risk for developing human papillomavirus (HPV)-induced cancers such as oropharyngeal cancer, cervical cancer, anogenital cancer and anal cancers [5–10]. However, the cellular and molecular mechanisms explaining the correlation between increased susceptibility of HPV-associated diseases and HIV-induced immune suppression remain largely unknown.

The oropharyngeal cancers are a subset of head and neck cancers (HNCs) which account for approximately 4% of all the cancers in the United States. The incidence of disease was twice as high in men than in women in 2015 [11]. The oropharyngeal cancers, in their clinically distinct form of squamous cell carcinoma, are commonly detected in HIV patients with a higher number of lifetime oral sexual partners, immunosuppression, smoking, and current tobacco use. The causal role of oral HPV infection is supported by substantial molecular and cellular evidence [5–7, 12].

Recent studies suggested that the interaction between HIV and HPV might be responsible for the increased risk of cervical cancers [13, 14]. Similarities in risk factors for the acquisition of HIV and HPV infections, such as high-risk sexual behavior, multiple sexual partners, and disease-related immunosuppression makes the demarcation between the HPV and HIV-associated malignancies challenging. It has been hypothesized that HIV patients who have been infected for a long period of time have a higher prevalence of oral HPV infection and subsequently are at a higher risk for HPV-associated HNCs [15]. HIV-induced immunosuppression also increases the risk of HPV-associated cancer. Other factors that can increase the risk of severe HPV infections in HIV patients include immune senescence, aging, impaired immune response to HPV, and direct interaction between the two viruses [13, 14, 16]. The immunological changes caused by HIV create a permissive immune environment, thereby decreasing the overall immune responses against HPV. However, the mechanisms by which HIV-induced reduction in CD4+ T cell levels impairs the immune response against HPV or other pathogens remain largely unknown.

Besides immunological factors, interactions at the molecular level between HIV genes *tat*, *rev* and *vpr* and HPV have also been reported. Multiple studies indicate an up-regulation of HPV oncogenic genes (*E6* and *E7*) expression by *tat* [14, 17, 18]. *Tat* increases HPV shedding which suggests that HIV infection may contribute to the pathogenesis of HPV-associated disease by molecular interactions through *tat* [16].

Currently, there are two commercially available HPV preventive vaccines, the quadrivalent human papillomavirus vaccine (QHPV) (*Gardasil;* Merck) against HPV types 6, 11, 16 and 18 and a bivalent vaccine (*Cervarix*, Glaxosmithkline) against HPV types 16 and 18. Both vaccines are known to be 98–100% effective against HPV types 16 and 18 [19]. Recent studies demonstrated the efficacy of the anti-HPV vaccine *Cervarix* in HIV-infected-oral-HPV-negative patients where more than 90% of patients produced high antibody titers against HPV [20]. However, more data is needed to address cross-reactivity between the induced antibody and other HPV strains [21]. Moreover, additional efficacy trials of QHPV in HIV infected individuals are needed to properly determine the correlation between vaccines dose, timing and protection against all HPV genotypes in immunocompromised HIV patients. The mechanistic investigation of co-infection scenario is experimentally challenging. The disadvantages of interrupted treatment of HIV and limited efficacy for the HPV vaccine against all the HPV strains further adds to the complexity. The limited clinical information about treatment and prevention options against HPV in an HIV/HPV co-infection has lead us to an alternative mode of investigation.

To gain new insights into the underlying mechanistic interactions between HIV and HPV, in an HIV/HPV co-infection we developed a mathematical model of HIV and HPV interactions. Building on previous models of HIV [22] and HPV [23] single infections, this novel model captures the molecular interactions between HIV and HPV due to *tat* and the effect of progressive depletion of CD4+ T cell due to HIV infection. Using the model, we aim to investigate why the prevalence of oral HPV infection is increased in HIV-infected individuals. We demonstrate how the dynamics of HPV changes in an HIV/HPV co-infection when the patient undergoes combined antiretroviral therapy. The findings can be used to further advance our understanding of the mechanisms underlying oral immune plasticity. Lastly, modeling can help propose new hypotheses for reversing residual inflammation in individuals following the start of cART and guide clinical practice.

## Methods

### Mathematical model of HIV infection

We model the interaction between HIV and CD4+ T cells as in [22, 24]. Briefly, we consider the interaction between three populations: i) target CD4+ T cells (*T*), ii) productively infected CD4+ T cells (*I*), and iii) HIV (*V)*. Target cells are produced at rate *s*, die at rate *d*, and become productively infected at rate *β* proportional to the interaction between target cells and the virus. Infected cells produce *N*_*1*_ virions throughout their lifetime, which are released through bursting, and die at rate *δ*. The virus is cleared at a rate *c*_*1*_ per day. The following system of ordinary differential equations (ODE) represents these dynamics:

$$\begin{array}{l} \;\frac{\text{dT}}{\text{dt}}=s-\text{dT}-\beta \text{TV},\\ \;\frac{\text{dI}}{\text{dt}}=\beta \text{TV}-\delta I,\\ \frac{\text{dV}}{\text{dt}}=N_{1}\delta I-c_{1}V, \end{array}$$ (1)

with initial conditions *T(0)* = *T*_0,_ *I(0) = I*_*0*,_ and *V(0) = V*_*0*._

The effect of cART has been modeled as a reduction of the virus infectivity in the presence of reverse transcriptase inhibitors to *β* (1- ε_*RT*_*)* and a reduction in the production of infectious virions in the presence of protease inhibitors to *N*_*1*_(1- ε_*PI*_). Here 0 ≤ ε_*RT*_, ε_*PI*_ ≤ 1 are the drug efficacies [24, 25].

The model in the presence of cART becomes:

$$\begin{array}{l} \frac{\text{dT}}{\text{dt}}=s-\text{d}T-(1-\varepsilon_{\text{RT}})\beta \text{TV},\\ \;\frac{\text{dI}}{\text{dt}}=(1-\varepsilon_{\text{RT}})\beta TV-\delta I,\\ \frac{\text{dV}}{\text{dt}}=(1-\varepsilon_{\text{PI}})N_{1}\delta I-c_{1}V, \end{array}$$ (2)

with initial conditions *T*(0) = *T*_1,_ *I*(0) = *I*_*1*,_ and *V*(0) = *V*_*1*_. Note that models (1) and (2) do not take into account the HIV latent reservoirs in the form of resting long-lived memory CD4+ T cells with integrated HIV in their genome [26].

### Mathematical model of HPV infection

We model HPV in-host dynamics as in [23]. We consider the interaction between four populations: i) HPV infected basal epithelial cells (*Y*_*1*_), ii) the HPV infected transit-amplifying cells, in the suprabasal epithelial layer (*Y*_*2*_), iii) HPV (*W)* and iv) HPV-specific cytotoxic T lymphocytes (CTL) (*E)*. We assume that *N*_*2*_ is the total concentration of epithelial cells at the beginning of HPV infection and the basal layer is formed of uninfected basal epithelial cells, targeted by HPV. Upon HPV infection, the basal epithelial cells become infected *Y*_*1*_ when HPV interacts with uninfected cells at rate *ψ*. We denote the difference *N*_*2*_ -*Y*_*1*_ as the concentration of uninfected epithelial cells. The basal infected cells, *Y*_*1*_ traverse up through the epithelial column and transform into *Y*_*2*_ cells, which move further into the suprabasal epithelial layer [27][28]. The *Y*_*2*_ cells become transit-amplifying cells which start assembling virions to be released at the surface [29, 30]. Therefore, both *Y*_*1*_ and *Y*_*2*_ cells are HPV infected cells but differentially located in the epithelial cell layer, wherein *Y*_*2*_ cells are assumed to have higher expression of the oncogenes *E6* and *E7* compared to *Y*_*1*_ [23]. For simplification, we assume that the uninfected cells and infected cells have an equal probability of interaction with the HPV virions irrespective of the spatial architecture of the tissue. A more generalized model which takes into account the infectivity and layer transition terms, or one which would consider spatial structures for epithelial cells in different layers requires extensive knowledge of numerous parameters, which are currently unknown. We assume that the infection is density dependent with *φ* representing the uninfected cell concentration where the infection is half-maximal. We assume that infected cell populations *Y*_*1*_ and *Y*_*2*_ differ in terms of the oncogene expression such that the *Y*_*2*_ (located in the suprabasal epithelial layer) have higher oncogene expression compared to *Y*_*1*_ cells (located in the basal epithelial layer) [31]. The rate of oncogene expression of the HPV type present in an infected cell, given by *ε* controls the conversion of *Y*_*1*_, into the transit-amplifying infected cells, *Y*_*2*._ Cells, *Y*_*2*_, grow at rate *rε*, proportional to their own density and die at rate *μ*. Due to higher expression of oncogenes, the transit-amplifying cells, *Y*_*2*_ divide more before death, compared to the basal infected cells *Y*_*1*_. Since, both infected cell population have an expression of oncogene, as in [23], both types of infected cells produce free virions (*W)*, at production rates *k*_*1*_ and *k*_*2*,_ that are released through bursting. For simplicity, we consider an equal virion production rate of *k*_*1*_ *= k*_*2*_ *= k*. The HPV virions are cleared at rate *c*_*2*_ [23]. The *c*_*2*_ clearance rate captures the antibody clearance rate implicitly.

The clearance of HPV in the infected cells, is associated with a successful immune response that includes the trigger of innate immune responses targeted against the virions released from the surface as well as infected cells [30]. In addition to the innate immune responses, the HPV-specific CTLs recruited during the adaptive immune response aid in the elimination of the infected basal cells [32]. Here, we assume that, after encountering transit-amplifying infected cells *Y*_*2*_, effector cells specific to HPV, *E*, expand with a maximum per capita rate *ω* and carrying capacity *K*. This carrying capacity is an addition to the original work [23]. In the current model, the CTL response *E* is initiated only by *Y*_*2*_ cells which have higher oncogene *E6 expression* [33] [34] compared to *Y*_*1*_.

We disregard the differential CTL response against the infected cell populations and consider that HPV-specific CTL population *E* kills both classes of infected cells at the same rate *a*, since both infected cells populations express oncogenes *E6* and *E7* [30][32]. Additionally, the model does not take into account the virus specific gene expression at any particular epithelial site. Finally, the functional differences in *E6* and *E7*, which are major determinants of HPV pathogenicity between HPV types [35], are also neglected.

The following system of differential equations represents these dynamics:

$$\begin{array}{l} \frac{{dY}_{1}}{\text{dt}}=\psi W\frac{N_{2}-Y_{1}}{\varphi +N_{2}-Y1}-{\varepsilon Y}_{1}-{\mu Y}_{1}-{\text{aY}}_{1}E,\\ \frac{{\text{dY}}_{2}}{\text{dt}}={\varepsilon Y}_{1}+r\varepsilon Y_{2}-{\mu Y}_{2}-{\text{aY}}_{2}E,\\ \;\frac{\text{dW}}{\text{dt}}=\mu k(Y_{1}+Y_{2})-c_{2}W,\\ \;\frac{\text{dE}}{\text{dt}}={\omega Y}_{2}E(1-\frac{E}{K}), \end{array}$$ (3)

with initial conditions *Y*_*1*_(0) = *Y*_10,_ *Y*_*2*_(0) = *Y*_*20*,_ *W*(0) = *W*_*0*_ and *E*(0) = *E*_*0*._

### Co-infection Model

#### The effect of *tat* protein

HIV-protein *tat*, secreted from HIV-infected intraepithelial immune cells, is known to play an important role in the disruption of epithelial tight junctions, thereby facilitating the entry of HPV into the mucosal epithelium [36]. We model the *tat-*induced increased likelihood of HPV infection through increasing the total available epithelial cells from *N*_*2*_ to *N*_*2*_ (1+*pV*), where (*p)* is the effect of *tat* protein secreted by an HIV virion (*V)*, given in Eq (4). The term (1+*pV*) incorporates the HIV-associated epithelial disruption as one of the major underlying mechanisms that increases the susceptibility of epithelial cells to the HIV/HPV co-infection [36].

$$\frac{{dY}_{1}}{\text{dt}}=\psi W\frac{(1+\text{pV})N_{2}-Y_{1}}{\varphi +(1+\text{pV})N_{2}-Y1}-{\varepsilon Y}_{1}-{\mu Y}_{1}-{\text{aY}}_{1}E.$$ (4)

#### The effect of immunosuppression

In HIV-infected individuals, the loss of CD4+ T cells leads to consecutive loss of CD4+ T cell-mediated immune responses against other pathogens such as HPV. Naïve CD4+ T cells are depleted in mucosal tissues in all the stages of HIV infection [26, 37], and progressive decline of CD4+ T cells affects the differentiation process of naïve CD4+ T cells into the different subsets. Such a subset, Th1, is known to play a major role in immune responses against HPV [34] through induction of cell-mediated immunity in the presence of IL-2, IL-12 and IFN-γ cytokines [38].

To model the decrease in the availability of CD4+ T cell population due to HIV; and the subsequent effect of such loss on HPV-specific CTL (*E)* responses, we assume that the carrying capacity of the *E* population decreases in an immunosuppressed patient. In particular, we represent *K* as the carrying capacity of CTLs and thus the maximum *E* population. We make *K* a function of CD4+ T cell population, such that *K* is given by, *K = K(T)* = *bT*, where *T* are the uninfected CD4+ T cells in the model (1). When *T* decreases during the progressive loss of CD4+ T cells, *T*, *K(T)*, the maximum carrying capacity decreases at a linear rate. We assume that the CTL carrying capacity is directly proportional to the amount of CD4+ T cells. Other modeling options, such as a T dependent source with a death term were explored, however the maximum proliferation term *K(T)* best explained the homeostatic mechanistic behavior of the CTLs.

**Fig 1:**
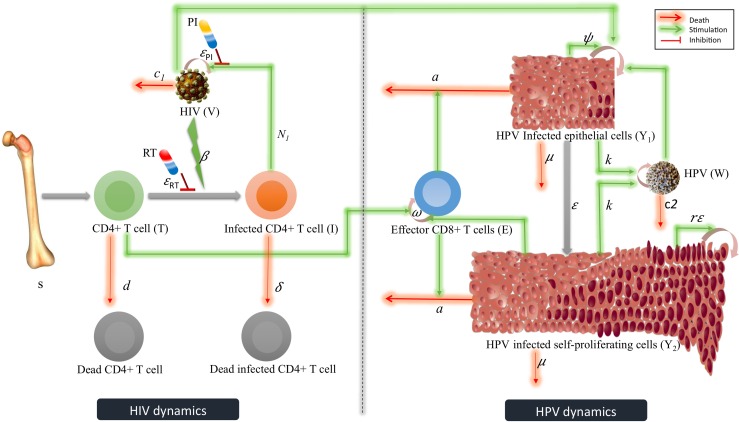
HIV HPV Diagram. A diagram for the co-infection model (5). The left side of the figure represents the HIV dynamics wherein the interaction between target CD4+ T cells (*T*), productively infected CD4+ T cells (*I*) and HIV (*V*) are shown. The figure also includes the effect of reverse transcriptase (*RT*) and protein inhibitor (*PI*) (shown by red line—inhibition). The right side of the figure represents the HPV dynamics wherein the interaction between infected basal cells (*Y*_*1*_), suprabasal transit-amplifying cells (*Y*_*2*_), HPV specific (*E*) cells and HPV (*W*) are shown. The systems biology markup language (SBML) compliant network of interactions between HIV (*V*) and HPV (*W*) is created using CellDesigner [40] (S1 Fig).

The co-infection model becomes (see Fig 1):

$$\begin{array}{l} \;\frac{\text{dT}}{\text{dt}}=s-\text{dT}-\left(1-\varepsilon_{\text{RT}}\right)\beta \text{TV},\\ \;\frac{\text{dI}}{\text{dt}}=\left(1-\varepsilon_{\text{RT}}\right)\beta \text{TV}-\delta I,\\ \;\frac{\text{dV}}{\text{dt}}=\left(1-\varepsilon_{\text{PI}}\right)N_{1}\delta I-c_{1}V,\\ \frac{dY_{1}}{\text{dt}}=\psi W\frac{\left(1+\text{pV}\right)N_{2}-Y_{1}}{\varphi +\left(1+\text{pV}\right)N_{2}-Y1}-\varepsilon Y_{1}-\mu Y_{1}-{\text{aY}}_{1}E,\\ \frac{{\text{dY}}_{2}}{\text{dt}}=\varepsilon Y_{1}+r\varepsilon Y_{2}-\mu Y_{2}-{\text{aY}}_{2}E\\ \;\frac{\text{dW}}{\text{dt}}=\mu k\left(Y_{1}+Y_{2}\right)-c_{2}W,\\ \;\frac{\text{dE}}{\text{dt}}=\omega Y_{2}E\left(1-\frac{E}{K\left(T\right)}\right), \end{array}$$ (5)

with initial conditions *T(0)* > 0, *I*(*0*) > 0, *V*(*0*) > 0, *Y*_*1*_(*0*) = *Y*_10,_ *Y*_*2*_(*0*) = *Y*_*20*,_ *W*(*0*) = *W*_*0*_ and *E*(*0*) = *E*_*0*_ where *t =* 0 is the time of co-infection.

The complete SBML compliant model was deposited in BioModels [39] and assigned the identifier MODEL 1605030001.

## Results

### Analytical results

Analytically, we can find a necessary condition for the HPV infection to be cleared (data in S1 File). We assume that we have a chronically infected HIV subject who reached steady state values ($\bar{T}$, $\bar{I}$, $\bar{V}$). Under these conditions, the carrying capacity for population E becomes $\bar{K}=b\bar{T}$. System (5) reduces to:

$$\begin{array}{l} \frac{{dY}_{1}}{\text{dt}}=\psi W\frac{(1+p\bar{V})N_{2}-Y_{1}}{\varphi +(1+p\bar{V})N_{2}-Y1}-{\varepsilon Y}_{1}-{\mu Y}_{1}-{\text{aY}}_{1}E,\\ \frac{{\text{dY}}_{2}}{\text{dt}}={\varepsilon Y}_{1}+r\varepsilon Y_{2}-{\mu Y}_{2}-{\text{aY}}_{2}E,\\ \;\frac{\text{dW}}{\text{dt}}=\mu k(Y_{1}+Y_{2})-c_{2}W,\\ \;\frac{\text{dE}}{\text{dt}}={\omega Y}_{2}E(1-\frac{E}{\bar{K}}). \end{array}$$ (6)

Then, HPV will clear when:

$$\frac{\psi k\mu (1+p\bar{V})N_{2}}{c_{2}(\varphi +(1+p\bar{V})N_{2})}<\frac{\left(\varepsilon +\mu +\text{aE})(-\varepsilon r+\text{aE}+\mu \right)}{\left(\varepsilon +\mu +\text{aE}-r\varepsilon \right)},$$ (7)

where *E* is any CTL level (data in S1 File). Biologically, this means that when the product between HPV infection rate and the HPV production rate (in the presence of HIV) is less than the combined effect of effector cells and natural death rate of HPV, clearance of HPV will be observed.

### Numerical results

Using the co-infection model (5), we numerically simulated disease scenarios in order to understand the dynamics of HPV infection in a co-infected individual. A recent clinical trial has investigated the effect of HIV in HPV infection in the presence and absence of combination antiretroviral therapy [41]. The levels of oral HPV DNA in the co-infected patients, which was monitored for 24 weeks after the start of cART, remained elevated throughout therapy. To determine the possible mechanisms of HPV persistence, we investigate models (5) and (6) for the relative contributions of co-infection factors: *tat*, as given by *pV*, and immunosuppression as given by *K*(*T*).

#### Parameter values

Parameter values from previously published studies are utilized here, as follows. Equilibrium values for HIV RNA per ml and HIV-specific uninfected CD4+ T cells per ml were reported in an HIV/HPV co-infection study to be $\bar{V}$ = 4.8x10^4^ virions per ml and $\bar{T}$ = 3.3x10^5^ cells per ml [41]. Since the patient is in a chronic HIV steady state, we derive $\bar{I}$, *β* and *s* from steady state conditions $\bar{I}=\frac{c1\bar{V}}{N1\delta }$, $\beta =\frac{c_{1}}{N_{1}\bar{T}}$ and $s=d\bar{T}+\bar{T}\bar{V}$, to be $\bar{I}$ = 2.4x10^3^ cells per ml, *β =* 1.5x10^-7^ ml per cells per day and *s =* 5.6x10^3^ cells per ml per day. The remaining parameters are summarized in Table 1.

**Table 1:** Parameters.

Parameter	Value	Description	Reference
*s*	5.6 x 10^3^ cells ml^-1^ day^-1^	CD4+ T cell recruitment rate	See text
*β*	1.5 x 10^−7^ ml virions^-1^ day^-1^	HIV infection rate	See text
*d*	0.01 day^-1^	Uninfected CD4+ T cell death rate	[42]
*δ*	1 day^-1^	Infected CD4+ T cells death rate	[43, 44]
*N*_*1*_	467 virions cells^-1^	HIV burst size	[45]
*c*_*1*_	23 day^-1^	HIV clearance rate	[46]
*ε_RT_*	varied	Reverse transcriptase efficacy	See text
*ε_PI_*	varied	Protease inhibitor efficacy	See text
$\bar{T}$	3.2 x 10^5^ cells ml^-1^	Uninfected CD4+ T cells at equilibrium	[41]
$\bar{I}$	2.4 x 10^3^ cells ml^-1^	Infected CD4+ T cells at equilibrium	See text
$\bar{V}$	4.8 x 10^4^ virions ml^-1^	HIV at equilibrium	[41]
*N*_*2*_	10^4^ cells	Total concentration of epithelial cells	[23]
*φ*	10^6^ cells	Epithelial cell concentration for which infection is half maximal	[23]
*Ψ*	0.0067 cells virions^-1^ day^-1^	HPV infection rate	[23]
*μ*	0.048 day^-1^	Epithelial cell death rate	[23]
*r*	0.1	Transit-amplifying cells recruitment rate	[23]
*ε*	varied	Oncogenic expression	See text
*ω*	10^−3^ cell^-1^ day^-1^	CTL expansion rate	[23]
*K*	varied	CTL carrying capacity	See text
a	0.01 day^-1^ cells^-1^	CTL killing rate	[23]
*k*	1000 virions cells^-1^	HPV burst size	[23]
*c*_*2*_	0.05 day^-1^	HPV clearance rate	[23]

We assume that the *tat-*effect, given by $p\bar{V}$, ranges between zero and 20, to account for up to a 20-fold increase in the target epithelial cell population due to co-infection. The immunosuppression factor, given by $K(\bar{T})=b\bar{T}$ ranges between $K(\bar{T})$ = 35 cells and $K(\bar{T})$ = 1 cell to account for changes in available CTL concentrations between an HPV infection and HIV/HPV co-infection. Lastly, the oncogene expression *ε* ranges from zero to one.

#### The viral dynamics of HPV infected individuals

**Fig 2:**
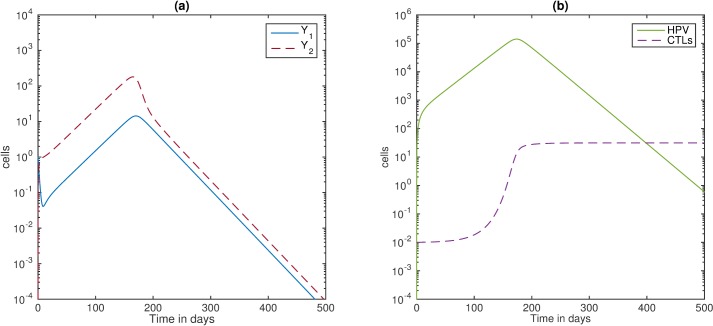
HPV infection. (a) Infected basal cells *Y*_*1*_ (blue solid line) and suprabasal, transit-amplifying cells, *Y*_*2*_ (red dashed line); (b) HPV *W* (green solid line) and CTL *E* (purple dashed line) for *ε* = 0.5 per day and $K(\bar{T})$ = 35 cells. All the other parameters are listed in Table 1.

We first study the dynamics of HPV infection in the absence of HIV, as given by model (6) with $\bar{I}$ = $\bar{V}$ = 0 cells per ml, and $\bar{T}$ = 10^6^ cells per ml. We let *ε* = 0.5 per day, $K(\bar{T})$ *=* 35 cells, *b* = 3.5x10^-5^ and the other parameters are listed in Table 1. Under these assumptions, model (6) predicts HPV and CTL levels similar to those in [23]. In particular, HPV reaches a maximum of 1.4x10^5^ copies at day 174 and eventual clearance (see Fig 2, panel b, green solid line). The CTL expansion is delayed by 80 days, and reaches an equilibrium value of 27 cells by day 240 (see Fig 2, panel b, purple dashed line). Transit-amplifying cells, *Y*_*2*_ with high oncogenic gene expression, are 12-times higher than cells with low oncogenic expression, *Y*_*1*_ (see Fig 2, panel a). This result is dependent on the oncogene expression rate *ε* (not shown).

#### The viral dynamics of HIV/HPV co-infected individuals

We start by assuming that the *tat* effect leads to the doubling of available target epithelial cells, *i.e.* $N_{2}(1+p\bar{V})=2N_{2}$. We then account for HIV induced immunosuppression in an HIV/HPV co-infected individual, by changing $K(\bar{T})$ as follows. We have shown in the previous section that an HIV-naïve individual has a CTL carrying capacity $K(\bar{T})$ = 35 cells, where $\bar{T}$ = 10^6^ CD4+ T cells per ml and *b* = 3.5x 10^−5^. We keep the *b* = 3.5x10^-5^ and decrease the $\bar{T}$ number to i) $\bar{T}$ = 5x10^5^ cells per ml, corresponding to average chronic HIV CD4+ T cell numbers [47]; ii) $\bar{T}$ = 3.3x10^5^ cells per ml as in the HIV/HPV co-infection study [41]; and iii) $\bar{T}$ = 2x10^5^ cells per ml, corresponding to AIDS.

**Fig 3:**
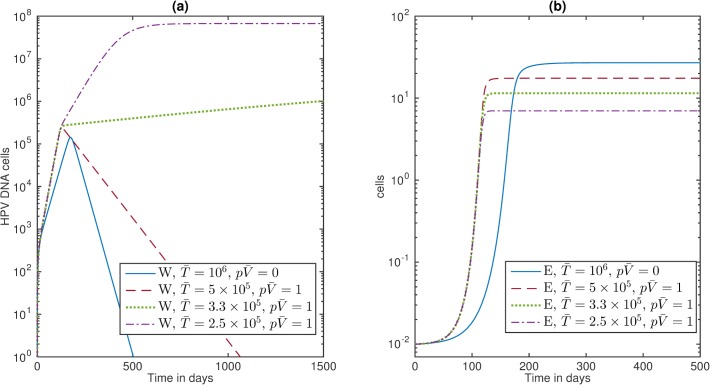
HIV/HPV co-infection. (a) HPV *W* and (b) CTL *E* as given by model (6) for *ε* = 0.5 per day, parameters are listed in Table 1, and $\bar{T}$ = 10^6^ cells per ml (blue solid lines); $\bar{T}$ = 5x10^5^ cells per ml (red dashed lines); $\bar{T}$ = 3.3x10^5^ cells per ml (green dotted lines); and $\bar{T}$ = 2x10^5^ cells per ml (purple dashed-dotted lines).

Under these assumptions and parameters in Table 1, model (6) predicts HPV clearance in cases (i) and HPV persistence in cases (ii) and (iii). In case (i), HPV levels reaches a maximum of 2.4x10^5^ copies at day 128 and clears by day 1050. In cases (ii) and (iii), HPV reaches steady state values of 3.5x10^6^ and 6.7x10^7^ DNA cells after 20 and 2.1 years, respectively (see Fig 3, panel a). CTL levels decrease to 17.5, 11.5 and 7 cells per ml for cases (i), (ii) and (iii), respectively (see Fig 3, panel b).

**Fig 4:**
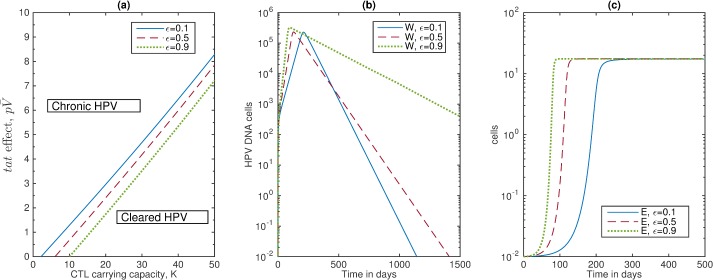
Varying oncogene expression rates. (a) Bifurcation diagram showing cleared *W* (area below the curve) versus chronic *W* (area above the curve) as the *tat* effect $p\bar{V}$ and CTL carrying capacity $K(\bar{T})$ vary. Here, the criterion for HPV clearance is given by Eq (7); (b) HPV *W;* and (c) CTL *E* as given by model (6) for parameters listed in Table 1 and *ε =* 0.1 (blue solid lines), *ε =* 0.5 (red dashed lines), and *ε =* 0.9 (green dotted lines).

To determine the relative contributions of the *tat-*effect and immunosuppression in the transition between HPV clearance and HPV persistence, we derived a bifurcation diagram showing the asymptomatic dynamic of HPV as given by model (6) when both $p\bar{V}$ and $K(\bar{T})$ are varied. As expected, an increase in the available epithelial cells requires a larger CTL population for the clearance to occur (see Fig 4, red dashed lines). In particular, if the *tat* effect is increased to 100% such that $\left(1+p\bar{V}\right)$ = 2, then the CTL carrying capacity has to be *K* >11.9 cells for clearance to occur. Moreover, a carrying capacity as low as $K(\bar{T})$ = 7 cells is enough to ensure HPV clearance in the HIV-naïve case (80% lower than the considered base value of $K(\bar{T})$ = 35 cells).

#### Changing oncogene expression rates

We have considered that the oncogenic expression is *ε* = 0.5. In an HIV-naïve host, this corresponds to transit-amplifying cells, *Y*_*2*_ exceeding the infected basal cells, *Y*_*1*_ by 12-times (see Fig 2, panel a). In the mathematical model from [23], the authors showed that in an HIV-naïve, HPV-unvaccinated individual, a decrease in the oncogenic expression *ε* leads to a slower growth of *Y*_*1*_, *Y*_*2*_ and *W*, a delayed and weak CTL response *E* and, consequently, a delayed HPV clearance. To determine whether this effect is carried over in an HIV/HPV co-infected individual, we compared clearance regions for *ε* = 0.1 per day, *ε* = 0.5 per day and *ε* = 0.9 per day for varying $p\bar{V}$ and $K(\bar{T})$ values (see Fig 4, panel a). We find that the clearance regions (defined as the area under the curve) are higher for low *ε* values, similar to the results from an HIV-naïve patient [23].

To determine the timing of clearance, we fixed the *tat*-effect to $\left(1+p\bar{V}\right)$ = 2 and the CTL carrying capacity to $K(\bar{T})$ = 17.5 cells and determined the changes in *W* and *E* dynamics for various values of *ε* (see Fig 4, panels b and c). We find that HPV levels are slightly higher for high oncogenic expression, *ε*, and they take significantly longer to get cleared (see Fig 4, panel b). This happens in spite of the fact that CTL levels grow faster for high oncogenic expression (see Fig 4 panel c).

#### The effect of cART on an HIV/HPV co-infection

A recent trial has investigated the dynamics of oral HPV DNA in HIV/HPV co-infected individuals undergoing cART. They found that 28% of the co-infected individuals had a persisting infection with at least one of the HPV genotypes. Moreover, 42% of co-infected individuals experienced either a persisting infection with the same genotype, or an acquired infection with a different genotype 24 weeks after the start of therapy [41]. We use model (5) to determine the *tat* effect *pV*, CTL numbers *K*(*T*), and oncogenic expression *ε* that can explain this observation.

The patients in [41] have $\bar{T}$ = 3.3x10^5^ uninfected CD4+ T cells per ml and $\bar{V}$ = 4.8x10^4^ virions per ml at day *t* = 0, when cART begins. We assume that the drug efficacies are *ε*_RT_ = 0.95, *ε*_PI_ = 0.5 and the oncogenic factor is *ε* = 0.5. If the co-infection with HPV is not included *i*.*e*. $p\bar{V}$ = 0 and *K(*$\bar{T}$*)* = 35 cells, then HIV RNA levels decrease to below limit of detection (of 50 copies per ml) in 6.5 days. CD4+ T cell concentration increases to a maximum of 5.6x10^5^ cells per ml by day 329 (161 days after the end of the study).

**Fig 5:**
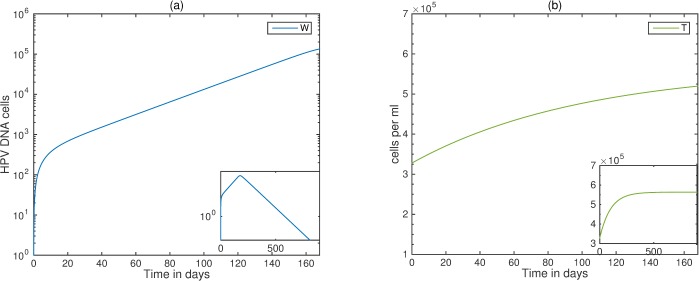
HIV/HPV dynamics when cART and HPV infection coincide. (a) HPV *W*; (b) CD4+ T cells *(T)* as given by model (5) under cART. Here, *ε* = 0.5, *ε*_RT_ = 0.95, *ε*_PI_ = 0.5, and all other parameters are listed in Table 1. Initial conditions are *T*_*0*_ = 3.3x10^5^ cells per ml, *I*_*0*_ = 2.4x10^5^ cells per ml, *V*_*0*_ = 4.8x10^4^ virions per ml, *Y*_*10*_ = 1 cells, *E*_*0*_ = 0.01 cells, *Y*_*20*_ = *W*_*0*_ = 0, and *t* = 0 is the start of cART. Over the first 24 weeks HPV persists (panel a), and in the long term HPV is cleared (zoomed out panel a).

We next add co-infection into our model and apply it to the setup of the oral co-infection trial [41]. If *pV* = 1 and *K*(*T*) = 11.5 cells (corresponding to CD4+ T cell concentration of $\bar{T}$ = 3.3x10^5^ per ml), then HPV is cleared under the cART conditions *ε*_RT_ = 0.95 and *ε*_PI_ = 0.5. The timing of clearance depends on two factors: the HPV stage and the level of CD4+ T cells at the start of cART. If HPV infection occurs at the same time as the start of cART, then the HPV levels increase to a peak value of 1.4x10^5^ DNA cells and stay elevated throughout the 24 weeks of the study (see Fig 5, panel a). HPV starts to decay at day 180 (see Fig 5, panel a, zoomed out graph) when CD4+ T cells reached 5.2x10^5^ cells per ml (see Fig 5, panel b) which is the low level of CD4+ T cell counts for healthy individuals.

**Fig 6:**
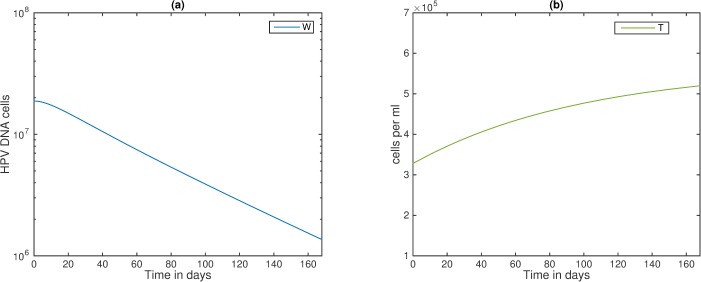
HIV/HPV dynamics under chronic HPV at the start of cART. (a) HPV *W*; (b) CD4+ T cells *T* as given by model (5) under cART. Here, *ε* = 0.5 per day, *ε*_*RT*_ = 0.95, *ε*_*PI*_ = 0.5, and all other parameters are listed in Table 1. Initial conditions are *T*_*0*_ = 3.3x10^5^ cells per ml; *I*_*0*_ = 2.4x10^5^ cells per ml; *V*_*0*_ = 4.8x10^4^ virions per ml; *Y*_*10*_ = 3.2x10^3^ cells; *Y*_*20*_ = 1.6 x10^4^ cells; *W*_*0*_ = 1.8x10^7^ virions; *E*_*0*_ = 0.01 cells, and *t* = 0 is the start of cART. Moreover, we found two instances when HPV infection stays chronic in the presence of cART, namely strong drug efficacy, *ε*_*RT*_ = 0.95 and *ε*_*PI*_ = 0.5, and AIDS level CD4+ T cells, $\bar{T}$ ≤ 1.7×10^5^ cells per ml; and, inefficient drug therapy, *ε*_*RT*_ = 0.2 and *ε*_*PI*_ = 0 and intermediate CD4+ T cell levels $\bar{T}$ ≤ 2.6×10^5^ cells per ml.

In contrast, if HPV infection reached its chronic state at the start of cART, then cART helps to initiate HPV decay by day 8 (see Fig 6, panel a), when the CD4+ T cell population is still low, i.e., $\bar{T}$ = 3.5×10^5^ cells per ml (see Fig 6, panel b). It is worth noting that cART removes HIV, and consequently the *tat* effect, but it does not control how fast CD4+ T cells rebound.

**Fig 7:**
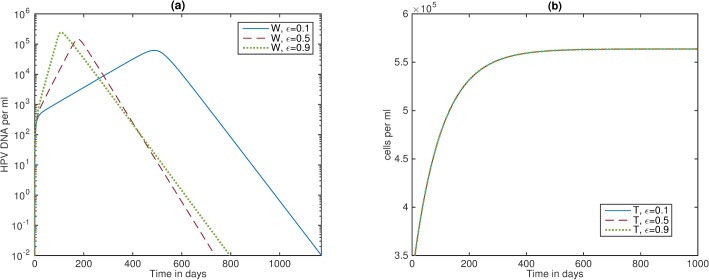
Effect of oncogene expression rates and cART on HIV/HPV co-infections. (a) HPV *W*; (b) CD4+ T cells *T* as given by model (5) under cART. Here, *ε* = 0.1 (blue solid line); *ε* = 0.5 (red dashed line); *ε* = 0.9 (green dotted line), *ε*_RT_ = 0.95, *ε*_PI_ = 0.5, and all other parameters are listed in Table 1. Initial conditions are *T*_*0*_ = 3.3x10^5^ cells per ml, *I*_*0*_ = 2.4x10^5^ cells per ml, *V*_*0*_ = 4.8x10^4^ virions per ml, *Y*_*10*_ = 1 cells, *E*_*0*_ = 0.01 cells, *Y*_*20*_ = *W*_*0*_ = 0 and *t* = 0 is the start of cART.

Lastly, we investigated how the dynamics of HPV infection in a co-infected individual undergoing cART change with the oncogenic expression *ε*. We found that HPV levels stay high throughout the first 24 weeks of cART, but are eventually cleared for all levels of oncogenic expression (see Fig 7, panel a). This is due to the fact that the dynamics of CD4+ T cells are not affected by the oncogenic expression (see Fig 7, panel b), and, in all cases, the patients return to healthy CD4+ T cell levels. HPV has lower peak levels but longer time until clearance for low oncogenic expression, *ε* = 0.1 (see Fig 7, panel a, blue solid line) compared to high oncogenic expression *ε* = 0.9 (see Fig 7, panel a, green dotted line).

**Fig 8:**
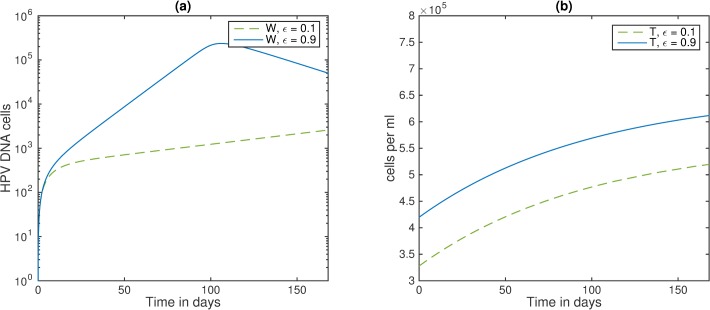
Effect of CD4+ T cell levels on HIV/HPV co-infections. (a) HPV W; (b) CD4+ T cells, *T*, as given by model (5) under cART. Here, *ε* = 0.1 (green dashed line), *ε* = 0.9 (blue solid line), *ε*_*RT*_ = 0.95, *ε*_*PI*_ = 0.5, and all other parameters are listed in Table 1. Initial conditions are T_0_ = 3.3x10^5^ (green dashed line) or T_0_ = 4.5x10^5^ (blue solid line) cells per ml, I_0_ = 2.4x10^5^ cells per ml, V_0_ = 4.8x10^4^ virions per ml, *Y*_*10*_ = 1 cells, *E*_*0*_ = 0.01 cells, *Y*_*20*_ = *W*_*0*_ = 0 and t = 0 is the start of cART.

An intriguing finding in [41] showed higher frequency of HPV DNA in individuals with the strongest rebound in absolute CD4+ T cell count post cART [41]. To investigate possible underlying mechanisms explaining this unexpected finding, we considered two virtual patients: patient 1 has a rebound to 6.5x10^5^ cells per ml CD4+ T cell count as in [41], and patient 2 has a rebound to 5.6x10^5^ cells per ml. We further assume that patient 1 has high oncogenic expression level *ε* = 0.9 per day, and patient 2 has low oncogenic expression level *ε* = 0.1 per day. This increase in oncogene expression leads to higher HPV DNA production in patient 1 (see Fig 8 panel a, solid blue vs dashed green line) in spite of its better cART outcome (see Fig 8, panel b, solid blue versus dashed green lines).

We investigated the coinfection model using the full model from [23] and found that it gives similar results (data in S2 File) to the coinfection model (6) and the differences are negligible (Fig A in S2 Fig and Fig B in S2 Fig).

## Discussion

The model presented here is a mechanistic ordinary differential equation (ODE)-based model that studies the dynamical interaction between the host and two virus populations: HIV and HPV. The model is aimed towards determining the mechanistic interactions leading to HPV clearance or persistence in HIV/HPV co-infected individuals post cART, and increased risk of HPV infection in HIV infected individuals as reported clinically [41] [48] [15, 36]. Indeed, a recent study reported an increased prevalence of oral HPV infection in an HIV-infected cohort, where HPV DNA levels in the patients were not reduced following treatment with antiretroviral therapy [41]. This result is corroborated by other studies, suggesting that HPV may be present chronically in oral sites among HIV infected individuals undergoing combination antiretroviral therapy [49, 50].

To address the possible interactions leading to HPV persistence, we highlighted specific scenarios presenting an increased persistence of HPV due to the permissive immune environment created in an HIV-infected individual. Our model predicted that among the HIV infected individuals, those who had CD4+ T cells corresponding to average chronic HIV CD4+ T cell levels were more likely to clear HPV than those who had the CD4+ T cell levels reported in the co-infection clinical study [41], or those who had CD4+ T cell levels corresponding to AIDS. These results are dependent on the oncogenic expression levels, with HPV DNA levels increasing and taking a longer time to clear for high oncogenic expression levels. Interestingly, for high oncogenic expressions, HPV clearance is delayed despite the faster expansion of CTL levels. This is due to an increase in the amount of HPV transit-amplifying cells.

We used the model to study the impact of cART on HPV persistence in HIV/HPV co-infected individuals and compared the findings with those from the clinical study [41], which showed that 28% of the co-infected patients had at least one detectable HPV DNA genotype 12–24 weeks after the start of cART. Our mechanistic model predicts that, for the median CD4+ T cell levels in [41], HPV levels decrease in co-infected individual receiving cART by 24 weeks. In addition, our model showed persistence of HPV DNA levels between 12–24 weeks post cART in the patients with the highest CD4 T cell rebound, as in [41]. The HPV DNA levels may be higher in the patients with the higher CD4 T cell rebound after cART if these patients have a higher HPV oncogenic expression. However, for these individuals with restored high levels of CD4+ T cells post cART, we predict clearance for HPV levels at 48 weeks. These findings, which differ from those in [41], can be explained by the absence of consideration for latent HIV reservoirs and latent HPV infection. Thus, reactivation of the latent HIV reservoirs and latent HPV infection is an integral part of immune reconstitution in the co-infected individuals and is guaranteed to impact HIV/HPV co-infection dynamics. Some studies have investigated the prevalence of anal human papillomavirus infection in HIV-infected patients receiving long-term cART. Piketty et al, showed that the incidence of anal cancer was higher in HIV-infected patients particularly in MSM (men who have sex with men) and the incidence of anal HPV infections did not reduce despite the increased CD4+ T cell count following cART [51], suggesting that cART- associated immune restoration does not play a role in reduction of the incidence of anal cancers [52]. Other studies have analyzed the effects of HIV and cART on HPV persistence and cervical squamous intraepithelial lesions. Blitz et al reported higher rate of acquisition and reduced clearance of oncogenic high-risk HPV types among HIV positive women. The study reported that cART improved clearance of high risk HPV type other than oncogenic HPV type 16/18 [53]. Interestingly, the findings from the study are in accordance with the findings from our model, wherein the oncogenic high risk HPV types persisted in the patients restored to high CD4+ T cell level post 24 weeks of cART.

Our study predicts that the timing of viral clearance is determined by the timing of cART compared to the timing of HPV co-infection, as well as the CD4 T cell level. When cART is started shortly after HPV infection, HPV will expand and will not be controlled in the 24 weeks of cART as described for the 28% cases in [41]. This can be explained by the fact that CTL expansion and control of HPV is delayed due to both recruitment and size limitation observed when CD4+ T cell reconstitution following cART is delayed. If, however, the cART starts at the peak anti-HPV CTL response, then HPV is cleared as soon as CD4+ T cell reconstitution allows for the adequate CTL levels to control HPV infection. Thus, our findings support the conclusion that increased risk of infection [48] due to immunosuppression may play a role in the reduced clearance of HPV. Specifically, we showed that chronic HPV levels were maintained at- i) AIDS level CD4+ T cell count and ii) inefficient drug therapy, *ε*_*RT*_ = 0.2 and *ε*_*PI*_ = 0 with intermediate CD4+ T cell levels such that $\bar{T}$ ≤ 2.6×10^5^ cells. These results provide further evidence to support the findings from other studies that show immunosuppression plays a role prior to cancer diagnosis [48] and facilitates HPV related head and neck carcinogenesis [15,30]. The results from our model are independent of the molecular effects induced by *tat*, which are removed when HIV is removed, and therefore do not influence HPV clearance. Finally, our results show that when HPV infection occurs at the same time as the start of cART, HPV levels increase and stay elevated throughout the 24 weeks of study. The findings support previous reports that predicted an increased risk of oral HPV infection among immunosuppressed individuals, which can be explained by the reactivation or reacquisition of a previously acquired infection [48]. We further investigated the possibility of HPV persistence and found that weak cART or AIDS level CD4+ T cells, which do not rebound to high enough levels, are needed for HPV to remain chronic following cART. These results highlight the important role played by CD4+ T cells in the resolution and control of HPV infection. It also demonstrates the importance of cART in controlling molecular mechanisms such as *tat* and CD4+ T cell rebound. A finding from our model highlights the need of a higher carrying capacity in a co-infected individual for HPV clearance to occur. This suggests a potentially clinically testable hypothesis that—if a HIV/HPV co-infected individual is immunosuppressed, then they should be treated for immunosuppression first. Once the CD4+ T cell levels are restored, the individual can be treated against HPV. Moreover, if a person treated for HIV has restored CD4+ T cell levels, HPV treatment should be tailored towards type specific HPV. In particular, our simulation result show that individuals with high CD4+ T cell levels post cART, produce more HPV when their oncogenic expression is high, compared with a patient with a weaker CD4+ T cell restoration but a lower oncogenic expression. Hence, the treatment (vaccine) against the type specific HPV, may aid the viral clearance.

This work is one of the first HIV/HPV co-infection models that investigates the dynamics of HPV in HIV-infected individuals. The only other published HIV/HPV co-infection model is a transitional probability-based model [54], which was used to study the relationship between immune status and the probability of the type of HPV clearance in HIV infected patients. The model [54] showed that HPV clearance was mainly based on the level of CD4+ T cell count. The main difference between our findings compared to [54] stems from the fact that the current model takes into account that HPV clearance not only depends on the levels of CD4+ T cell count but also the stage of HPV infection. Our results are consistent with observed associations between immunosuppression and HPV persistence in several clinical studies [41] [48] [15, 36].

To understand the mechanisms responsible for progression to AIDS and for CD4+ T cell rebound following cART, it is necessary to understand the impact of HIV in the dynamics of each functional CD4+ T cell subsets [55–58]. Th17 cells [59] [60] appear to play a central role in the HIV pathogenesis. Th1/17 CD4+ T cells have a role as a long-term reservoir for HIV-1 infection, and are unaffected by cART [61]. Tfh serve as reservoirs of virus-infected cells [62] in the lymph node and peripheral blood. The central memory (T_CM_) are the major cellular reservoirs for HIV in the peripheral blood [63]. Furthermore, long-term cART is known to only partially restore the CD4+ T cell pool [64, 65] in the oral mucosal sites. Thus, investigating the T cell subsets involved in the HIV/HPV co-infection in our model would aid in better understanding of these mechanisms and the development of approaches for their therapeutic manipulations.

The present modeling study should be evaluated in the context of several limitations. First, it does not take into account the spatial structure of the epithelial tissue. A generalized model that takes in to account the infectivity and layer transition terms described by probabilities or one which would consider spatial structures for epithelial cells in different layers, requires extensive knowledge of numerous parameters, that are currently unknown. The second limitation concerns the immune clearance of lesions caused by HPV infections, which can lead to asymptomatic or latent infections with possibility of increased virion production upon immunosuppression [66–69]. This further necessitates the need for consideration of both the cellular environment and the site of infection which are important determinants of virus activity [35]. Third, the simplistic modeling approach employed in the current modeling study does not take into account the feedback from the HPV to HIV infection. Due to absence of feedback from HPV to HIV, we disregard the effects of cART induced immune reconstitution. Additionally, our study does not consider the activation of latent HIV reservoirs post cART, which may contribute to the emergence new HPV genotype infections in co-infected individuals as shown in [41].

The next step would be investigating the T cell subsets involved in the HIV/HPV co-infection in our model. Additionally, the findings of the modeling study and associated limitations guarantees and necessitates inclusion of latent T cell reservoirs which are involved in the activation of residual cART induced immune reconstitution. Furthermore, incorporating the immune activation in the T cells under the effect of cART during a HIV/HPV co-infection would corroborate the findings regarding the presence of new detectable HPV DNA.

In summary, we developed a novel mathematical and computational model of HIV/HPV co-infection and used it to present hypotheses for the mechanisms underlying HPV persistence in HIV/HPV co-infected individuals. Our model can be applied to studying interactions between HIV and other widespread microbes to gain a better mechanistic understanding, guide the rational for the design of clinical trials, and accelerate the path to safer and more effective vaccines and therapeutics. We use this study as an alternative approach to determining how overall CD4+ T cell levels influence HPV prognosis in an HIV-infected individual. Overall, a better understanding of the cell specificity of HIV infection integrated with the cellular environment in HPV infection would facilitate the development of more effective therapeutic strategies in HIV/HPV co-infections.

The model was deposited in BioModels [39] and assigned the identifier MODEL 1605030001.

## Supporting Information

S1 Fig Systems biology markup language (SBML) compliant network of interactions between HIV (V) and HPV (W) created using CellDesigner. The left side of the figure represents the HIV dynamics wherein the interaction between target CD4+ T cells (*T*), productively infected CD4+ T cells (*I*) and HIV (*V*) are shown. The figure also includes the effect of reverse transcriptase (*RT*) and protein inhibitor (*PI*) (shown by red line—inhibition). The right side of the figure represents the HPV dynamics wherein the interaction between infected basal cells (*Y*_*1*_), suprabasal transit-amplifying cells (*Y*_*2*_), HPV specific (*E*) cells and HPV (*W*) are shown.
(PDF)

S2 FigFull model comparison.**(**A) HIV/HPV co-infection model comparison. (a) HPV W and (b) CTL *E* as given by model (5), solid blue lines and model (26), dashed red lines, for *ε* = 0.5 per day, parameters are listed in Table 1 for different $\bar{T}$ levels- $\bar{T}$ = 10^6^ cells per ml (first row); $\bar{T}$ = 5x10^5^ cells per ml (second row); $\bar{T}$ = 3.3x10^5^ cells per ml (third row); and $\bar{T}$ = 2x10^5^ cells per ml (fourth row). **(B)** HIV/HPV dynamics when cART and HPV infection coincide. (a) HPV *W*; (b) CD4+ T cells *(T)* as given by model (5) solid blue lines and model (26) dashed red lines under cART. Here, *ε* = 0.5, *ε*_RT_ = 0.95, *ε*_PI_ = 0.5, and all other parameters are listed in Table 1 and *t* = 0 is the start of cART. Over the first 24 weeks HPV persists (panel a), and in the long term HPV is cleared (zoomed out panel a).(PDF)

S1 File Analytical results. (PDF)

S2 File Analysis of the coinfection model using the full model. (PDF)
