# Supplementary material for: Modeling the Mechanisms by Which HIV-Associated Immunosuppression Influences HPV Persistence at the Oral Mucosa
Source: PLoS One. 2017 Jan 6;12(1):e0168133. doi: 10.1371/journal.pone.0168133 (PMC5218576; doi:10.1371/journal.pone.0168133)
Supplement: S1 Fig — The left side of the figure represents the HIV dynamics wherein the interaction between target CD4+ T cells (T), productively infected CD4+ T cells (I) and HIV (V) are shown. The figure also includes the effect of reverse transcriptase (RT) and protein inhibitor (PI) (shown by red line—inhibition). The right side of the figure represents the HPV dynamics wherein the interaction between infected basal cells (Y1), suprabasal transit-amplifying cells (Y2), HPV specific (E) cells and HPV (W) are shown. (PDF) [file pone.0168133.s001.pdf]

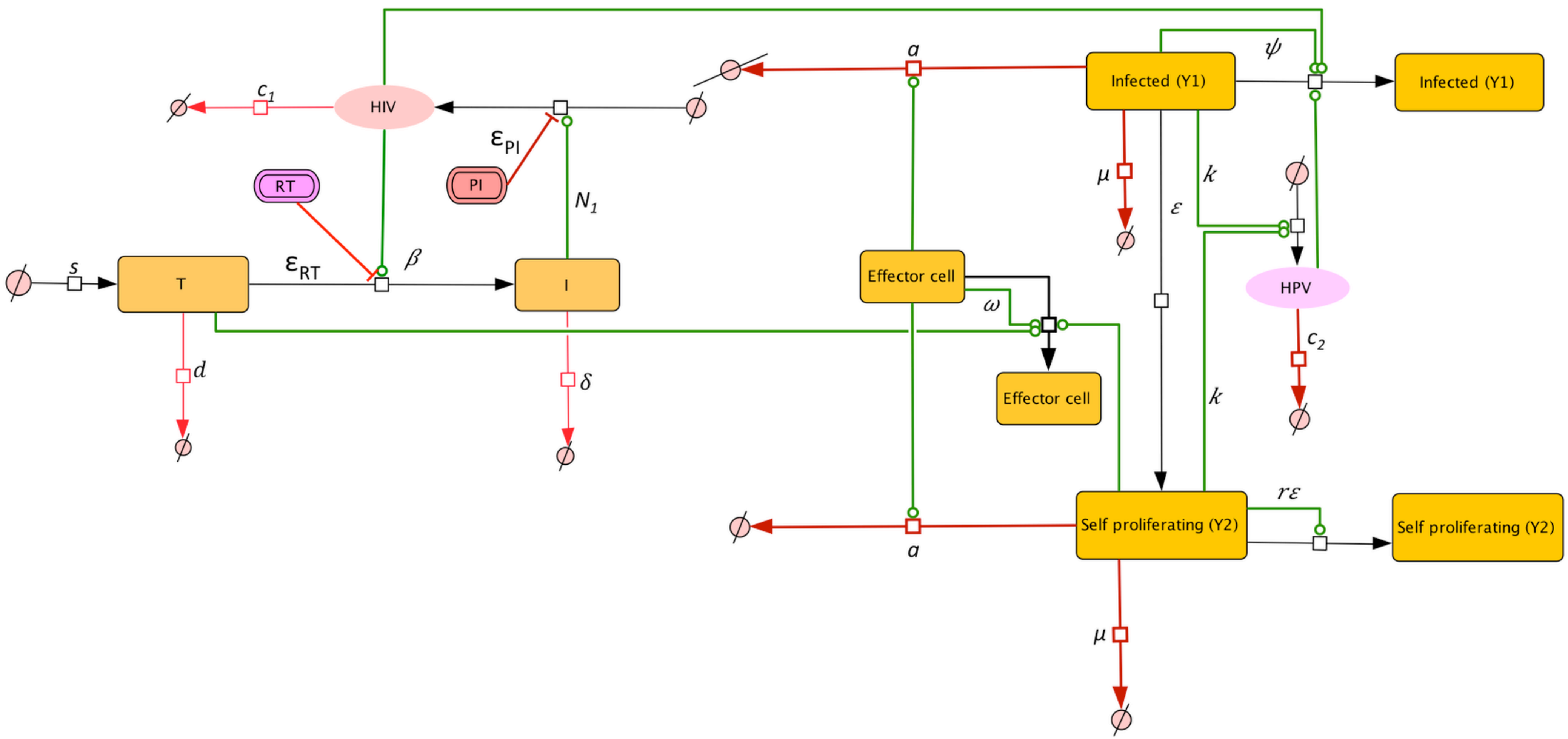

Fig. S1 Systems biology markup language (SBML) compliant network of interactions between HIV (*V*) and HPV (*W*) created using CellDesigner. The left side of the figure represents the HIV dynamics wherein the interaction between target CD4+ T cells (*T*), productively infected CD4+ T cells (*I*) and HIV (*V*) are shown. The figure also includes the effect of reverse transcriptase (*RT*) and protein inhibitor (*PI*) (shown by red line - inhibition). The right side of the figure represents the HPV dynamics wherein the interaction between infected basal cells (Y_1), suprabasal transit-amplifying cells (Y_2), HPV specific (*E*) cells and HPV (*W*) are shown.
