# Supplementary material for: Modeling the Mechanisms by Which HIV-Associated Immunosuppression Influences HPV Persistence at the Oral Mucosa
Source: PLoS One. 2017 Jan 6;12(1):e0168133. doi: 10.1371/journal.pone.0168133 (PMC5218576; doi:10.1371/journal.pone.0168133)
Supplement: S2 Fig — (A) HIV/HPV co-infection model comparison. (a) HPV W and (b) CTL E as given by model (5), solid blue lines and model (26), dashed red lines, for ε = 0.5 per day, parameters are listed in Table 1 for different T¯ levels- T¯ = 106 cells per ml (first row); T¯ = 5x10^5^ cells per ml (second row); T¯ = 3.3x10^5^ cells per ml (third row); and T¯ = 2x10^5^ cells per ml (fourth row). (B) HIV/HPV dynamics when cART and HPV infection coincide. (a) HPV W; (b) CD4+ T cells (T) as given by model (5) solid blue lines and model (26) dashed red lines under cART. Here, ε = 0.5, εRT = 0.95, εPI = 0.5, and all other parameters are listed in Table 1 and t = 0 is the start of cART. Over the first 24 weeks HPV persists (panel a), and in the long term HPV is cleared (zoomed out panel a). (PDF) [file pone.0168133.s002.pdf]

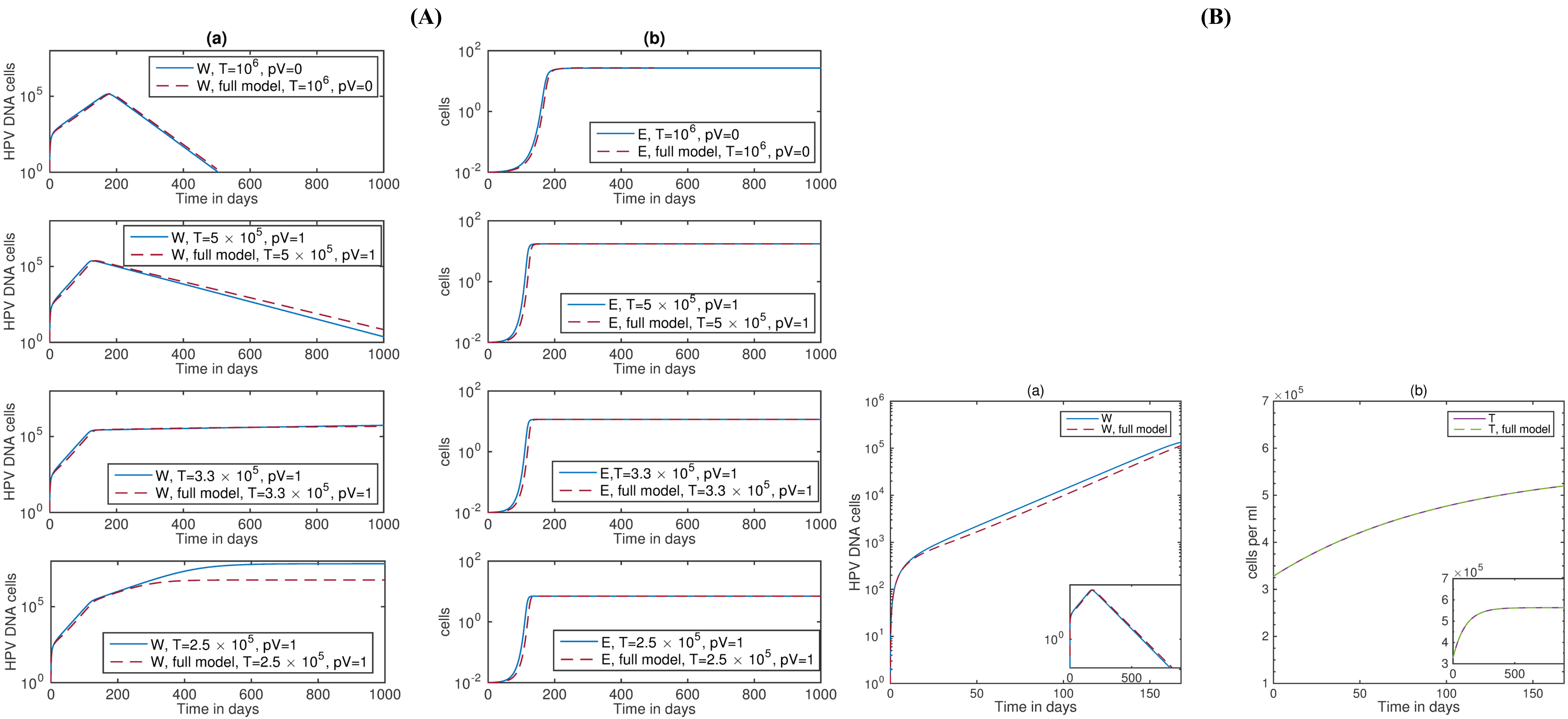

Fig. S2 Full model comparison. (A) HIV/HPV co-infection model comparison (a) HPV W and (b) CTL E as given by model (5), solid blue lines and model (26), dashed red lines, for $\varepsilon = 0.5$ per day, parameters are listed in Table 1 for different $\overline{T}$ levels- $\overline{T} = 10^6$ cells per ml (first row); $\overline{T} = 5 \times 10^5$ cells per ml (second row); $\overline{T} = 3.3 \times 10^5$ cells per ml (third row); and $\overline{T} = 2 \times 10^5$ cells per ml (fourth row). (B) HIV/HPV dynamics when cART and HPV infection coincide (a) HPV W; (b) CD4+ T cells *(T)* as given by model (5) solid blue lines and model (26) dashed red lines under cART. Here, $\varepsilon = 0.5$, $\varepsilon_{RT} = 0.95$, $\varepsilon_{PI} = 0.5$, and all other parameters are listed in Table 1 and $t = 0$ is the start of cART. Over the first 24 weeks HPV persists (panel a), and in the long term HPV is cleared (zoomed out panel a).
