# Supplementary material for: Modeling the Mechanisms by Which HIV-Associated Immunosuppression Influences HPV Persistence at the Oral Mucosa
Source: PLoS One. 2017 Jan 6;12(1):e0168133. doi: 10.1371/journal.pone.0168133 (PMC5218576; doi:10.1371/journal.pone.0168133)
Supplement: S1 File — (PDF) [file pone.0168133.s003.pdf]

S3 File. Analytical results

We analytically investigate the properties of the co-infection model (5) at a chronic HIV steady state. As described, our model only captures the effect HIV has on HPV dynamics and, hence, we can simplify model (5) to include the four HPV equations, and constant $\overline{T}$= 3.2x10^5 cells per ml, $\overline{I} = \frac{c_1\overline{V}}{N_1\delta}$ = 2.4x10^3 cells per ml and $\overline{V}$ = 4.8x10^4 virions per ml, *i.e.* ($\overline{T}, \overline{I}, \overline{V}$) are at a steady state. We also assume k_1=k_2=k as listed in Table 1. The reduced model is:

$$
\begin{aligned}
\frac{dY_1}{dt} &= \psi W \frac{(1+p\overline{V})N_2 - Y_1}{\varphi + (1+p\overline{V})N_2 - Y1} - \varepsilon Y_1 - \mu Y_1 - \mathrm{a}Y_1 E, \\
\frac{dY_2}{dt} &= \varepsilon Y_1 + r\varepsilon\, Y_2 - \mu Y_2 - \mathrm{a}Y_2 E, \\
\frac{dW}{dt} &= \mu(k_1 Y_1 + k_2 Y_2) - c_2 W, \\
\frac{dE}{dt} &= \omega Y_2 E\left(1 - \frac{E}{\overline{K}}\right).
\end{aligned}
\qquad (6)
$$

System (6) has three steady states: A virus free steady state,

$$S_0 = (0,0,0,E), \qquad (8)$$

a chronic immuno tolerant HPV steady state,

$$S_1 = (\widehat{Y_1}, \widehat{Y_2}, \widehat{W}, 0), \qquad (9)$$

where,

$$\widehat{Y_1} = \frac{Acr(\varepsilon + \mu) + Bk\mu\psi(1-r)}{-k\psi\mu r + c\varepsilon r + c\mu r + k\mu\psi}, \qquad (10)$$

$$\widehat{Y_2} = \frac{-Acr(\varepsilon+\mu) + Bk\mu\psi(r-1)}{r(-k\psi\mu r + c\varepsilon r + c\mu r + k\mu\psi)}, \qquad (11)$$

$$\widehat{W} = \mu k \frac{Acr(\varepsilon+\mu)(r-1) - Bk\mu\psi(r-1)^2}{rc(-k\psi\mu r + c\varepsilon\, r + c\mu r + k\mu\psi)}, \qquad (12)$$

with A = $(1+p\overline{V})N_2$ and B $=\varphi+(1+p\overline{V})N_2$ and a chronic immuno competent HPV steady state,

$$S_2=(\overline{Y_1},\overline{Y_2},\overline{W},\overline{E}), \tag{13}$$

where,

$$\begin{aligned}\overline{Y_1}&=(A\overline{K}^2a^2c+A\overline{K}^2ac\mu-A\overline{K}ac\varepsilon\, r-B\overline{K}ak\mu\psi-B\overline{K}k\mu^2\psi+B\varepsilon k\mu\psi r\\&+A\overline{K}ac\varepsilon\mu+A\overline{K}ac+A\overline{K}c\varepsilon\mu+A\overline{K}c\mu^2-Ac\varepsilon^2r-Ac\varepsilon\mu r-B\varepsilon k\mu\psi)/(\overline{K}^2a^2c\\&+\overline{K}^2ac\mu-\overline{K}ac\varepsilon r-\overline{K}ak\mu\psi-\overline{K}k\mu^2\psi+\varepsilon k\mu\psi r+\overline{K}ac\varepsilon+\overline{K}ac\mu+\overline{K}c\varepsilon\mu\\&+\overline{K}c\mu^2-c\varepsilon^2r-c\varepsilon\mu r-\varepsilon k\mu\psi),\end{aligned} \tag{14}$$

$$\begin{aligned}\overline{Y_2}&=\varepsilon(A\overline{K}^2a^2c+A\overline{K}^2ac\mu-A\overline{K}ac\varepsilon r-B\overline{K}ak\mu\psi-B\overline{K}k\mu^2\psi+B\varepsilon k\mu\psi r\\&+A\overline{K}ac\varepsilon+A\overline{K}ac\mu+A\overline{K}c\varepsilon\mu+A\overline{K}c\mu^2-Ac\varepsilon^2r-Ac\varepsilon\mu r-B\varepsilon k\mu\psi)/(\overline{K}^3a^3c\\&+2\overline{K}^3a^2c\mu+\overline{K}^3ac\mu^2-2\overline{K}^2a^2c\varepsilon r-\overline{K}^2a^2k\mu\psi-2\overline{K}^2ac\varepsilon\mu r-2\overline{K}^2ak\mu^2\psi\\&-\overline{K}^2k\mu^3\psi+\overline{K}ac\varepsilon^2r^2+2\overline{K}a\varepsilon k\mu\psi r+2\overline{K}\varepsilon k\mu^2\psi r-\varepsilon^2k\mu\psi r^2+\overline{K}^2a^2c\varepsilon\\&+\overline{K}^2a^2c\mu+2\overline{K}^2ac\varepsilon\mu+2\overline{K}^2ac\mu^2+\overline{K}^2c\varepsilon\mu^2+\overline{K}^2c\mu^3-2\overline{K}a\varepsilon^2cr-2\overline{K}ac\varepsilon\mu r\\&-\overline{K}a\varepsilon k\mu\psi-2\overline{K}c\varepsilon^2\mu r-2\overline{K}c\varepsilon\mu^2r-\overline{K}\varepsilon k\mu^2\psi+c\varepsilon^3r^2+c\varepsilon^2\mu r^2+\varepsilon^2k\mu\psi r),\end{aligned} \tag{15}$$

$$\begin{aligned}\overline{W}&=[Ac(\overline{K}^2a^2+\overline{K}^2a\mu-\overline{K}a\varepsilon r+\overline{K}a\varepsilon+\overline{K}\varepsilon\mu+\overline{K}a\mu+\overline{K}\mu^2\\&-\varepsilon^2r-\varepsilon\mu r)-Bk\mu\psi(\overline{K}a+\overline{K}\mu+\varepsilon-r\varepsilon)\mu k](\overline{K}a+\overline{K}\mu-\varepsilon r\\&+\varepsilon)/c(\overline{K}^3a^3c+2\overline{K}^3a^2c\mu+\overline{K}^3ac\mu^2-2\overline{K}^2a^2c\varepsilon r-\overline{K}^2a^2k\mu\psi\\&-2\overline{K}^2ac\varepsilon\mu r-2\overline{K}^2ak\mu^2\psi-\overline{K}^2k\mu^3\psi+\overline{K}acr^2\varepsilon^2+2\overline{K}ak\varepsilon\mu\psi r\\&+2\overline{K}k\varepsilon\mu^2\Psi r-\varepsilon^2k\mu\psi r^2+\overline{K}^2a^2c\varepsilon+\overline{K}^2a^2c\mu+2\overline{K}^2ac\varepsilon\mu+2\overline{K}^2ac\mu^2\\&+\overline{K}^2c\varepsilon\mu^2+\overline{K}^2c\mu^3-2\overline{K}ac\varepsilon^2r-2\overline{K}ac\varepsilon\mu r-\overline{K}a\varepsilon k\mu\psi-2\overline{K}c\varepsilon^2\mu r\\&-2\overline{K}c\varepsilon\mu^2r-\overline{K}\varepsilon k\mu^2\Psi+c\varepsilon^2r^2+c\varepsilon^2\mu r^2+\varepsilon^2k\mu\psi r),\end{aligned} \tag{16}$$

$$\overline{E}=\overline{K}\,, \tag{17}$$

To investigate the asymptomatic stability of S_0, we compute the Jacobian for the model (6):

$$J = \begin{bmatrix} -\psi W \frac{\varphi}{(\varphi + (1+p\overline{V})N_2 - Y_1)^2} - \varepsilon - \mu - aE & 0 & \psi \frac{(1+p\overline{V})N_2 - Y_1}{\varphi + (1+p\overline{V})N_2 - Y_1} & -aY_1 \\ \varepsilon & r\varepsilon - \mu - aE & 0 & -aY_2 \\ \mu k & \mu k & -c_2 & 0 \\ 0 & \omega E(1 - \frac{E}{\overline{\overline{K}}}) & 0 & \omega Y_2 - 2\omega a Y_2 \frac{E}{\overline{\overline{K}}} \end{bmatrix}. \tag{18}$$

At S_0,

$$J(S_0) = \begin{bmatrix} -\varepsilon - \mu - E - \lambda & 0 & \Omega & 0 \\ \varepsilon & r\varepsilon - \mu - E - \lambda & 0 & 0 \\ \mu k & \mu k & -c_2 - \lambda & 0 \\ 0 & \omega E(1 - \frac{E}{\overline{\overline{K}}}) & 0 & -\lambda \end{bmatrix}, \tag{19}$$

where, $\psi \frac{(1+p\overline{V})N_2 - Y_1}{\varphi + (1+p\overline{V})N_2 - Y_1} = \Omega$ The corresponding characteristic equation is:

$$\begin{aligned} &(-\lambda)[\lambda^3 + \lambda^2(c_2 - \varepsilon r + 2aE + \varepsilon + 2\mu) + \lambda[c_2(\varepsilon + \mu + aE) \\ &+ c_2(-\varepsilon r + aE + \mu) - \Omega\mu k + (-\varepsilon - \mu - aE)(\varepsilon r - aE - \mu)] \\ &+ (\varepsilon + \mu + aE)c_2(\mu + aE - r\varepsilon) - \varepsilon\mu k\Omega] = 0. \end{aligned} \tag{20}$$

We know that there is a λ such that $\lambda = 0$.

By Routh-Hurwitz criterion, all other eigenvalues are negative when $a_1 > 0$, $a_3 > 0$ and $a_1 a_2 > a_3$,

where,

$$a_1 = c_2 \quad r + 2aE + \quad + 2 \quad , \tag{21}$$

$$a_2 = c_2(\varepsilon + \mu + aE) + c_2(-\varepsilon r + aE + \mu) - \Omega\mu k + (-\varepsilon - \mu - aE)(\varepsilon r - aE - \mu), \quad (22)$$

$$a_3 = (\varepsilon + \mu + aE)c_2(\mu + aE - r\varepsilon) - \varepsilon\mu k\Omega. \quad (23)$$

$a_1 > 0$ always since $r < 1$. $a_3 > 0$ when

$$\frac{\Omega\mu k}{c_2} < \frac{(\varepsilon + \mu + aE)(-\varepsilon r + aE + \mu)}{(\varepsilon + \mu + aE - r\varepsilon)}. \quad (24)$$

Finally, $a_1a_2 - a_3 > 0$ when $a_3 > 0$ (not shown).

Condition (24) translates to

$$\frac{\psi k\mu(1 + p\overline{V})N_2}{c_2(\varphi + (1 + p\overline{V})N_2)} < \frac{(\varepsilon + \mu + aE)(-\varepsilon r + aE + \mu)}{(\varepsilon + \mu + aE - r\varepsilon)}, \quad (25)$$

which means that when the HPV infection rate times the HPV production rate (in the presence of HIV) is less than the combined effect of effector cells and natural death rate of HPV, HPV will be cleared. The asymptotic stability of chronic states S_1 and S_2 is messy and will not be presented here.
