# Supplementary material for: Modeling the Mechanisms by Which HIV-Associated Immunosuppression Influences HPV Persistence at the Oral Mucosa
Source: PLoS One. 2017 Jan 6;12(1):e0168133. doi: 10.1371/journal.pone.0168133 (PMC5218576; doi:10.1371/journal.pone.0168133)
Supplement: S2 File — (PDF) [file pone.0168133.s004.pdf]

S2 File. Analysis of the coinfection model using the full model

We investigate the properties of the co-infection model using the large model developed in the original HPV work's supplementary material from Murall at al 2015. The extended model includes the population of uninfected basal epithelial cells, X, which are born at rate $\lambda(t)$ and die at rate μ. HPV, W, interacts with uninfected basal epithelial cells, X, at rate ψ to produce infected cells, Y_1. We assume that the infection is density dependent with φ representing the uninfected cell concentration where the infection is half-maximal. Below is the expanded co-infection model wherein we included the effect of *tat*, pV, with the birth of uninfected basal epithelium. The term $(1+pV)\lambda(t)$ accounts for the production of epithelial cells that are susceptible for HPV infection. The rest of the co-infection dynamics are described in the main text.

The expanded co-infection model becomes:

$$
\begin{aligned}
\frac{\mathrm{dT}}{\mathrm{dt}} &= s - \mathrm{dT} - (1-\varepsilon_{\mathrm{RT}})\beta\mathrm{TV},\\
\frac{\mathrm{dI}}{\mathrm{dt}} &= (1-\varepsilon_{\mathrm{RT}})\beta\mathrm{TV} - \delta I,\\
\frac{\mathrm{dV}}{\mathrm{dt}} &= (1-\varepsilon_{\mathrm{PI}})N_1\delta I - c_1 V,\\
\frac{\mathrm{dX}}{\mathrm{dt}} &= (1+pV)\lambda(t) - \mu X - \psi W\frac{X}{\varphi+X},\\
\frac{dY_1}{\mathrm{dt}} &= \psi W\frac{X}{\varphi+X} - \varepsilon Y_1 - \mu Y_1 - \mathrm{a}Y_1E,\\
\frac{\mathrm{dY_2}}{\mathrm{dt}} &= \varepsilon Y_1 + r\varepsilon Y_2 - \mu Y_2 - \mathrm{a}Y_2E,\\
\frac{\mathrm{dW}}{\mathrm{dt}} &= \mu k(Y_1+Y_2) - c_2W,\\
\frac{\mathrm{dE}}{\mathrm{dt}} &= \omega Y_2E\left(1-\frac{E}{K(T)}\right),
\end{aligned}
\tag{26}
$$

with initial conditions with initial conditions $T(0) > 0$, $I(0) > 0$, $V(0) > 0$, $X(0) > X_1$, $Y_1(0) = Y_{10}$, $Y_2(0) = Y_{20}$, $W(0) = W_0$ and $E(0) = E_0$ where $t = 0$ is the time of co-infection.

Since, *tat* is known to play an important role in the disruption of epithelial tight junctions, thereby

facilitating the entry of HPV into the mucosal epithelium [30]. We compare the dynamics of model (26) against those of main model *(5)* for the same *tat* effect $(1+p\overline{V})=2$.

When considering HIV induced immunosuppression in HIV/HPV co-infected individuals with different CD4+ T cells levels of i) $\overline{T}$ = 10^6 cells per ml, corresponding to a healthy patient ii) $\overline{T}$ = $5x10^5$ cells per ml, corresponding to average chronic HIV CD4+ T cell numbers [41]; iii) $\overline{T}$ = $3.3x10^5$ cells per ml as in the HIV/HPV co-infection study [35]; and iv) $\overline{T}$ = $2x10^5$ cells per ml, corresponding to AIDS; the extended model (26) has similar results as the reduced model (5) (see (A) in Fig. S2). Similarly, when we considered the effects of co-infection under the setup of the oral co-infection trial [35] for pV = 1 and $K(T)$ = 11.5 cells (corresponding to CD4+ T cell concentration of $\overline{T}$ = $3.3x10^5$ per ml) we found that both model (6) and (26) give similar results. In particular, HPV is cleared under cART conditions ε_{RT} = 0.95 and ε_{PI} = 0.5 and the timing of the clearance depends on two factors: the HPV stage and the level of CD4+ T cells at the start of cART (see (B) in Fig. S2).
